# Unusual morphological adaptations and processes associated with viviparity in an epizoic dermapteran

**DOI:** 10.1371/journal.pone.0195647

**Published:** 2018-04-25

**Authors:** Szczepan M. Bilinski, Mariusz K. Jaglarz, Ali Halajian, Waclaw Tworzydlo

**Affiliations:** 1 Department of Developmental Biology and Invertebrate Morphology, Institute of Zoology and Biomedical Research, Jagiellonian University, Gronostajowa 9, Krakow, Poland; 2 Department of Biodiversity, University of Limpopo, Sovenga, South Africa; Centre for Stem Cell Research, UNITED KINGDOM

## Abstract

Matrotrophic viviparity is a reproductive pattern in which offspring develop inside a female’s body which provides gas exchange and nutrients necessary for development. Besides placental mammals, structural and physiological aspects of matrotrophic viviparity are poorly characterized. In insects, the majority of species is oviparous, i.e. lay eggs, and viviparous reproduction has been reported only in 11 out of 44 orders, including earwigs (Dermaptera). Among dermapterans, matrotrophic viviparity has been reported in two epizoic subgroups: Arixeniidae and Hemimeridae. Here, we provide morphological evidence for distinct adaptations for this mode of viviparity in embryonic and maternal tissues in a representative of the latter subgroup, *Hemimerus talpoides*. Our study reveals a novel mechanism of maternal contribution to embryonic development which operates during oogenesis and involves characteristic modification of endoplasmic reticulum cisternae. Conspicuous and apparently inactive para-crystalline stacks of the endoplasmic reticulum are deposited in the oocyte cytoplasm and become activated during early embryonic development. Our analyses indicate additionally that in *Hemimerus*, transformed follicular/ovarian cells (on the mother’s side) and an evagination of the dorsal vessel (on the embryo’s side) converge to form a cephalic vesicle, structure analogous to a placenta. The cellular architecture of this unusual “cephalic placenta” points to its participation in an exchange of low molecular weight substances between a mother and developing embryo.

## Introduction

Two main reproductive patterns: oviparity and viviparity have been recognized among insects [1–4]. The most common is oviparity. Oviparous species lay eggs comprising reserve materials (yolk spheres, lipid droplets) that are used during embryonic development. In viviparous species, the embryos develop inside the female, which gives birth to live offspring. A wide spectrum of nutritional modes have been described in viviparous species. On one side of the spectrum, there are insects in which embryos retained inside the mother’s reproductive system rely entirely on reserve materials deposited in eggs (the lecithotrophic mode). On the other side of this spectrum, there are species in which the developing embryos are continuously supplied with nutrients from a mother into the developing embryo (the matrotrophic mode). However many species may combine both modes to a different degree (for further details see: [2, 3]).

Earwigs (Dermaptera) is a small insect order traditionally classified into three subgroups: two epizoic taxa, the Arixeniina and Hemimerina, and the free-living Forficulina [5–7]. The majority of dermapteran species is, like most insects, oviparous. In contrast, both epizoic groups are viviparous. It is believed that viviparity evolved in these groups to provide nymphs with an immediate contact with the host and to accelerate the life cycle [8].

**Fig 1:**
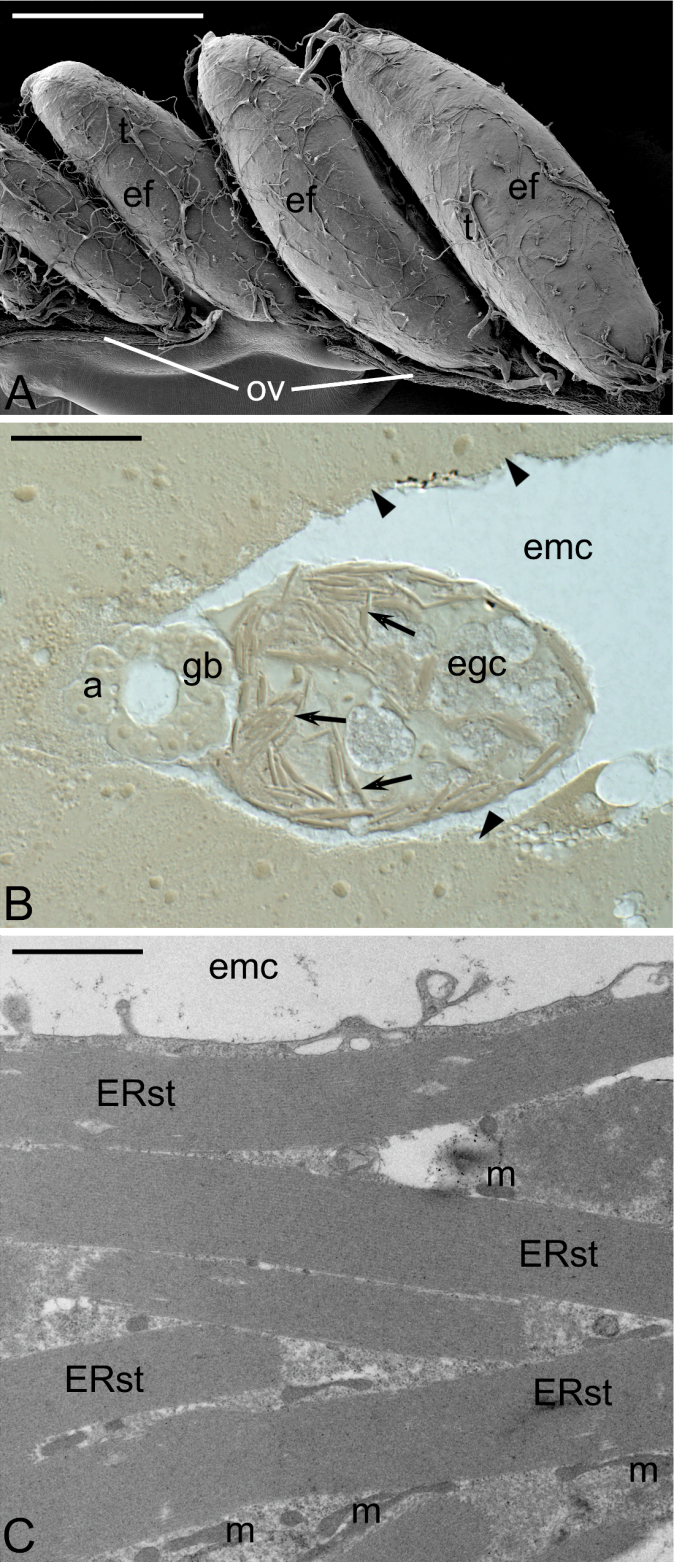
Embryonic follicle and early embryo. (A) SEM micrograph showing sequentially more advanced embryonic follicles (ef) attached to a lateral oviduct (ov), trachea (t). Scale bar: 1 mm. (B) Early embryo inside the embryonic cavity (emc); note a germ band (gb), amnion (a) and egg cytoplasm (egc) comprising stacks of ER (arrows); arrowheads indicate follicular epithelium surrounding the embryonic cavity; Nomarski interference contrast. Scale bar: 50 μm. (C) Fragment of the egg cytoplasm densely packed with highly organized ER stacks (ERst), mitochondria (m); TEM micrograph. Scale bar: 2 μm.

The Hemimerina live on Gambian pouched rats (*Cricetomus gambianus*). They feed on skin scales and fungi and are unable to survive outside the host [8]. The embryonic development of hemimerids has been analyzed only with classical histological methods more than a century ago by Heymons [9]. These studies have shown that *Hemimerus* embryos develop inside growing ovarian follicles, termed the embryonic follicles (Fig 1A). Such a mode of viviparity is referred to as intraovarian viviparity [3]. The lateral oviducts of the *Hemimerus* females are narrow, not modified into the uteri and apparently do not participate in the embryo nourishment. Our recent studies [10] confirmed these results and showed additionally that the mature *Hemimerus* oocytes reveal two peculiar characters: they are completely yolkless and not protected by multilayered egg envelopes. Instead, virtually entire oocyte cytoplasm (the ooplasm) is densely filled with unconventional organelles, i.e. the highly organized “para-crystalline” stacks of endoplasmic reticulum (ER) and translucent vacuoles that do not play any apparent role during oogenesis. It has been suggested, based on these findings that these organelles may function only after initiation of embryonic development [10].

The course of events during *Hemimerus* development has been reinterpreted by Hagan [11]. It transpires from that reinterpretation that the embryonic development of *Hemimerus* comprises two well separated phases: early and late. The early phase is highly modified, apparently related to the matrotrophic viviparity. The late phase follows the pattern typical for oviparous dermapterans, e.g. *Forficula* [12] except for the appearance of a peculiar, roughly spherical structure at the occipital region of the embryo head. This structure has been termed the cephalic vesicle by Hagan and believed to participate in the transfer of nourishments from the mother to the embryo [11].

Here we present results of detailed electron microscopic (EM) analyses of structures and processes that support matrotrophy in *Hemimerus talpoides*. We show that the para-crystalline stacks of the endoplasmic reticulum (ER) are segregated to the defined set of embryonic cells where they are activated and participate in protein syntheses. We also show, that the cephalic vesicle consists of both ovarian (maternal) and embryonic tissues and, therefore, can be regarded as a structure analogous to a placenta.

## Material and methods

### Ethics statement

Trapping of the rats was conducted as a part of this research. The rats were trapped with the assistance of a veterinarian with regard to legal regulations concerning wild species protection, and after insect collection all rats were released back into the wild. During conducting the research, no rats were injured or harmed. *Hemimerus* is not protected or endangered species. The research project was approved by authorities of the Lajuma Research Centre and the Limpopo Department of Environmental Affairs and Tourism (LEDET) (permit no. 0089-MKT001-00004).

### Animals

Specimens of *H*. *talpoides* were collected from the fur of Gambian pouched rats (*Cricetomys gambianus)* trapped in Lajuma Research Centre (Sautpansberg Mountain Range, Republic of South Africa, 23°02'15.4"S 29°26'35.2"E) [10]. Ten specimens of *H*. *talpoides* were used. The embryos or whole embryonic follicles in various developmental stages were manually dissected from adult females (using a stereomicroscope, Leica EZ4) and fixed in a mixture of 2% formaldehyde and 2.5% glutaraldehyde in 0.1 M phosphate buffer, pH 7.3.

### Light and transmission electron microscopy

Two of the fixed samples were rinsed and photographed under a Nikon SMZ1500 stereomicroscope (Nikon, Tokyo, Japan). The remaining 8 samples designed for light and transmission electron microscopy studies were postfixed in 2% osmium tetroxide and 0.8% potassium ferrocyanide for 30 min at 4°C. After dehydration in a series of ethanol and acetone the material was embedded in Glycid Ether 100 (Epon 812) resin (Serva, Heidelberg, Germany). Thick sections (2–3 μm thick) were photographed without any staining under the light microscope (Leica DMR; Leica, Heidelberg, Germany) equipped with Nomarski interference contrast. The semi-thin sections (0.7 μm thick) were stained with 1% methylene blue and examined under a light microscope (Leica DMR or Nikon Eclipse Ni; Nikon, Tokyo, Japan). The photographs of larger embryo fragments were composed by assembling several images using the Image Composite Editor software. Ultrathin sections (80 nm thick) were contrasted with uranyl acetate and lead citrate according to standard protocols and analyzed with a transmission electron microscope (TEM) (JEM 2100; JEOL, Tokyo, Japan) at 80 kV. For measurements Image J software was used.

### Scanning electron microscopy

For SEM, the material was fixed and postfixed as described above. After dehydration in a graded series of ethanol, the material was critical-point dried, coated with gold and examined with a scanning electron microscope (Hitachi S-4700; Hitachi, Tokyo, Japan) at 25 kV.

## Results and discussion

Our analysis revealed that early embryos of *Hemimerus talpoides* are substantially smaller than fully grown oocytes of this species. An estimated volume of such oocytes exceeds 13 × 10^−6^ mm^3^, whereas that of an early embryo attains only about 11 × 10^−7^ mm^3^. Thus, the volume decreases approximately 10 fold. Interestingly, such tremendous decrease in the embryo volume coincides with almost complete disappearance of translucent vacuoles observed in the cytoplasm of a fully grown oocytes [10].

Although early *Hemimerus* embryos are tiny and compact they comprise three clearly separated elements: the germ band (a proper embryo), amnion (an extraembryonic membrane) and a small cytoplasmic area comprising yolk nuclei and numerous highly organized stacks of the ER (Fig 1B). As according to Heymons and Hagan this area is homologous to the central yolk mass characteristic for embryos of oviparous insects [9–11], it will be termed the egg cytoplasm. During this early phase, the embryo floats and develops in a relatively large space (Fig 1B) encompassed by the follicular epithelium, surrounding the growing oocyte before fertilization [10]. This space will be referred to as the embryonic cavity. It is possible that the translucent vacuoles present in fully grown oocytes (Fig 3C in [10]) and almost absent in the egg cytoplasm (Fig 1B), are expelled from the developing embryo and contribute to the formation of this cavity and/or its content.

**Fig 2:**
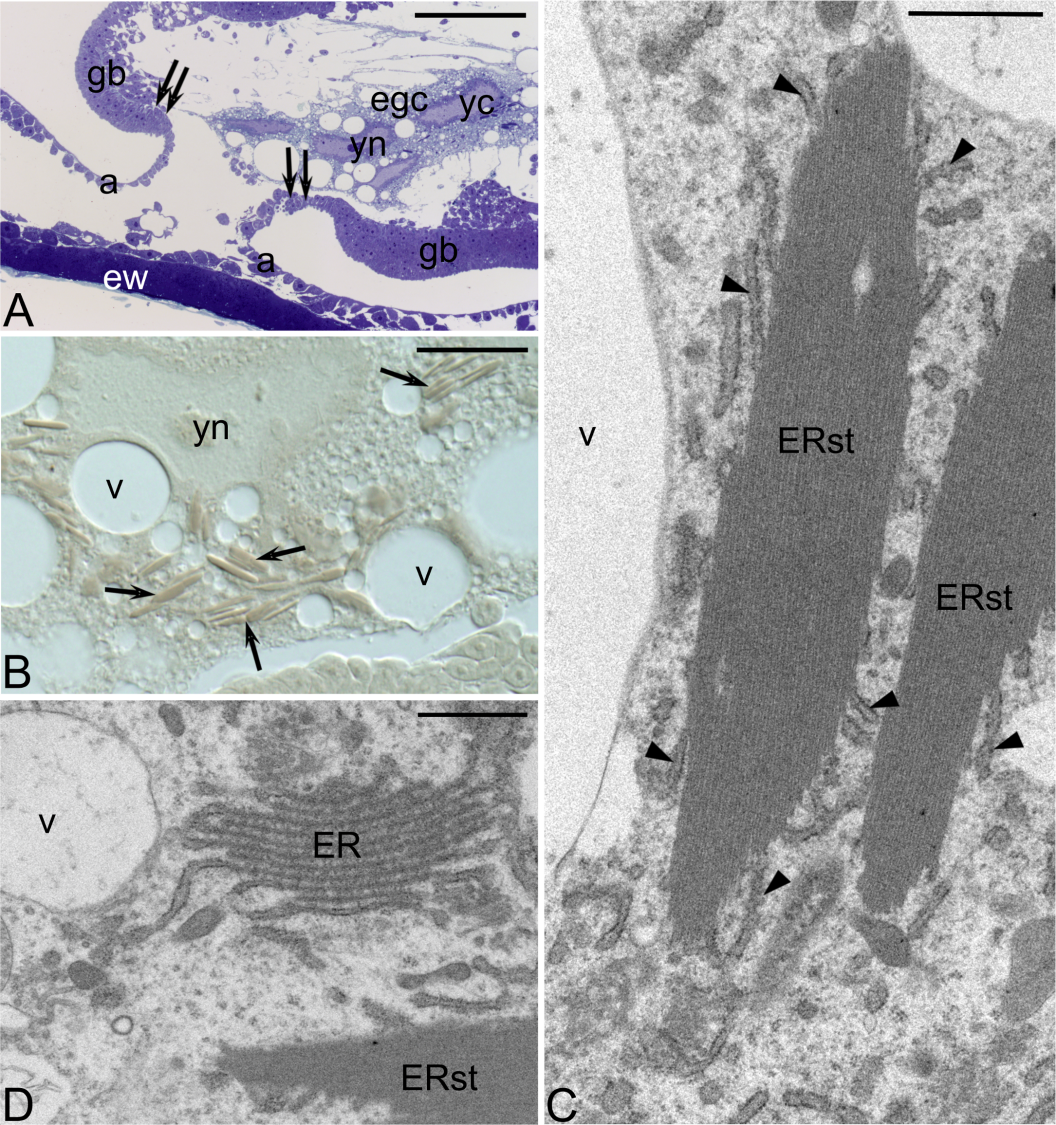
Embryo after dorsal flexion of the germ band. (A) Longitudinal section through a central region of the embryo; note the egg cytoplasm (egc) surrounded by the germ band (gb); yolk cell nucleus (yn), margins of the germ band are indicated by double arrows, the wall of the embryonic follicle (ew); semithin section stained with methylene blue. Scale bar: 100 μm. (B) Individual yolk cell comprising ER stacks (arrows); yolk cell nucleus (yn); Nomarski interference contrast. Scale bar: 50 μm. (C) TEM micrograph showing dispersing ER stacks; note that marginally located ER elements (arrowheads) are partly separated from the stack (ERst) and covered with ribosomes. Scale bar: 1 μm. (D) Canonical ER network in association with ER stack (ERst); vacuoles (v); TEM micrograph. Scale bar: 1 μm.

Our detailed analyses have shown that highly organized ER stacks present in the egg cytoplasm are morphologically identical to those present in the fully grown *Hemimerus* oocytes and consist of several parallel, closely adjoining ER cisternae (Fig 1C). The ER cisternae are not associated with ribosomes and therefore presumably do not participate in protein syntheses. Using semi-thin (0.7 μm) and thick (2–3 μm) serial sections, we have followed the fate of the ER stacks in subsequent developmental stages. We have found that these organelle assemblages remain morphologically unchanged till the stage, in which elongated germ band starts to bend dorsally and surrounds the egg cytoplasm (Fig 2A). At this stage, the yolk nuclei are relatively large and elongated (Fig 2A and 2B). They are immersed in the cytoplasm containing mitochondria, Golgi complexes, translucent vacuoles and dozens of the ER stacks (Fig 2B). In late germ band embryos, the ER stacks rearrange and gradually disperse (Fig 2C and 2D). Simultaneously, the superficially located membranes of each stack associate with ribosomes and translocate to the surrounding cytoplasm (Fig 2C). Eventually, the ER stacks are transformed into a canonical network of the rough endoplasmic reticulum (Fig 2D) that presumably participate in protein syntheses. This observation indicates that the transformation of the ER stacks leads to the formation of the powerful synthetic machinery in the cytoplasm of vitellophages. If so, the completely yolkless *Hemimerus* oocytes provide the embryo with the prefabricated synthetic machinery instead of supplying it with ready-to-use reserve materials, i.e. yolk proteins. To our knowledge, it is the first account of such unique process involving the transfer of inactive organelles from the oocyte to the embryo, and their activation during subsequent stages of the embryonic development. This is reminiscent of the deposition (localization) of dormant maternal gene products within the ooplasm, which are activated only after fertilization and commencement of the embryonic development [13].

**Fig 3:**
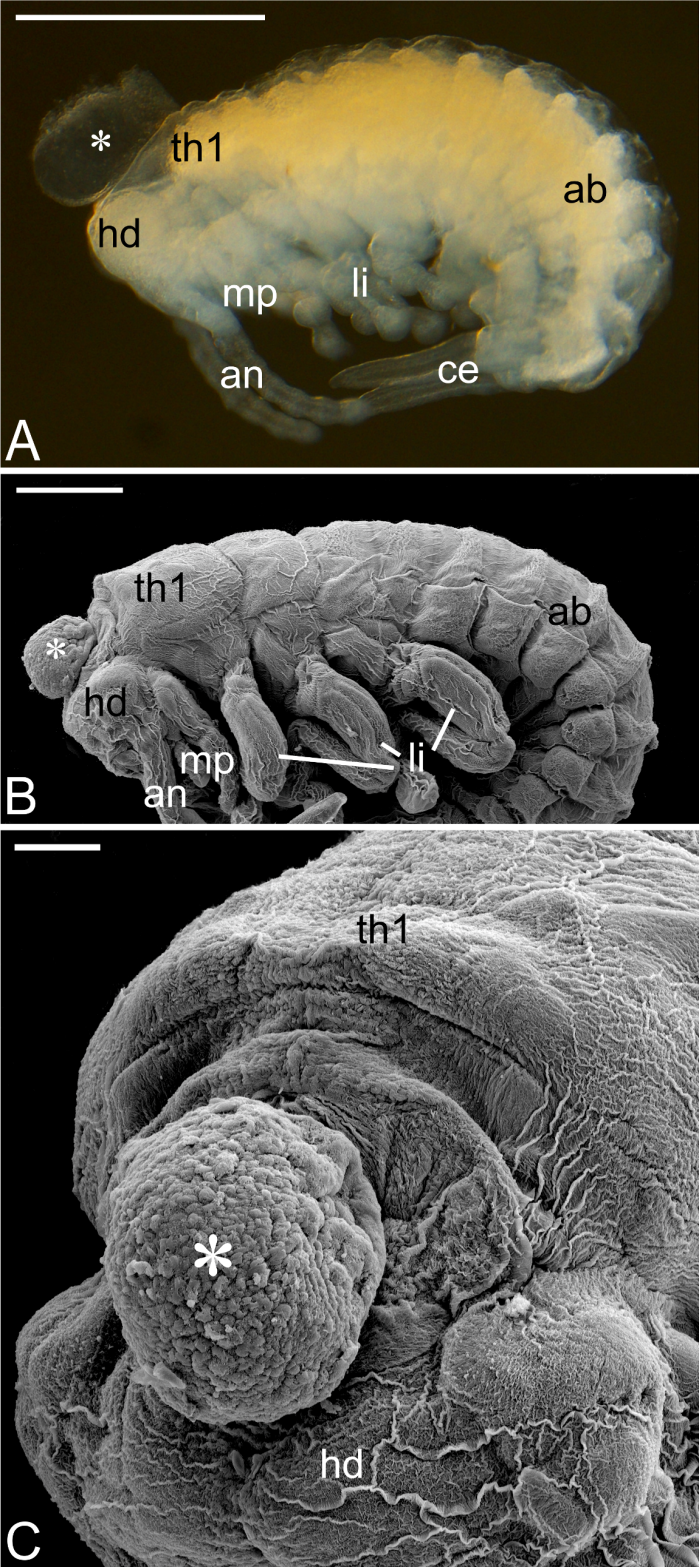
Late embryo. (A) Embryo soon after dorsal closure and katatrepsis (the stage during which the embryo assumes its final position on the egg surface); note the cephalic vesicle (asterisk) located between the head (hd) and the first thoracic segment (th1); photographed under a stereomicroscope. Scale bar: 1 mm. (B) SEM micrograph of a slightly older embryo; at this stage the cephalic vesicle (asterisk) is substantially smaller in relation to the head and thorax. Scale bar: 500 μm. (C) Front view of the same embryo as in b; note that the surface of the cephalic vesicle (asterisk) is covered with follicular cells. SEM micrograph. Scale bar: 100 μm. Abdomen (ab), antennae (an), cerci (ce), head (hd), first thoracic segment (th1), thoracic limbs (li), maxillary palps (mp).

**Fig 4:**
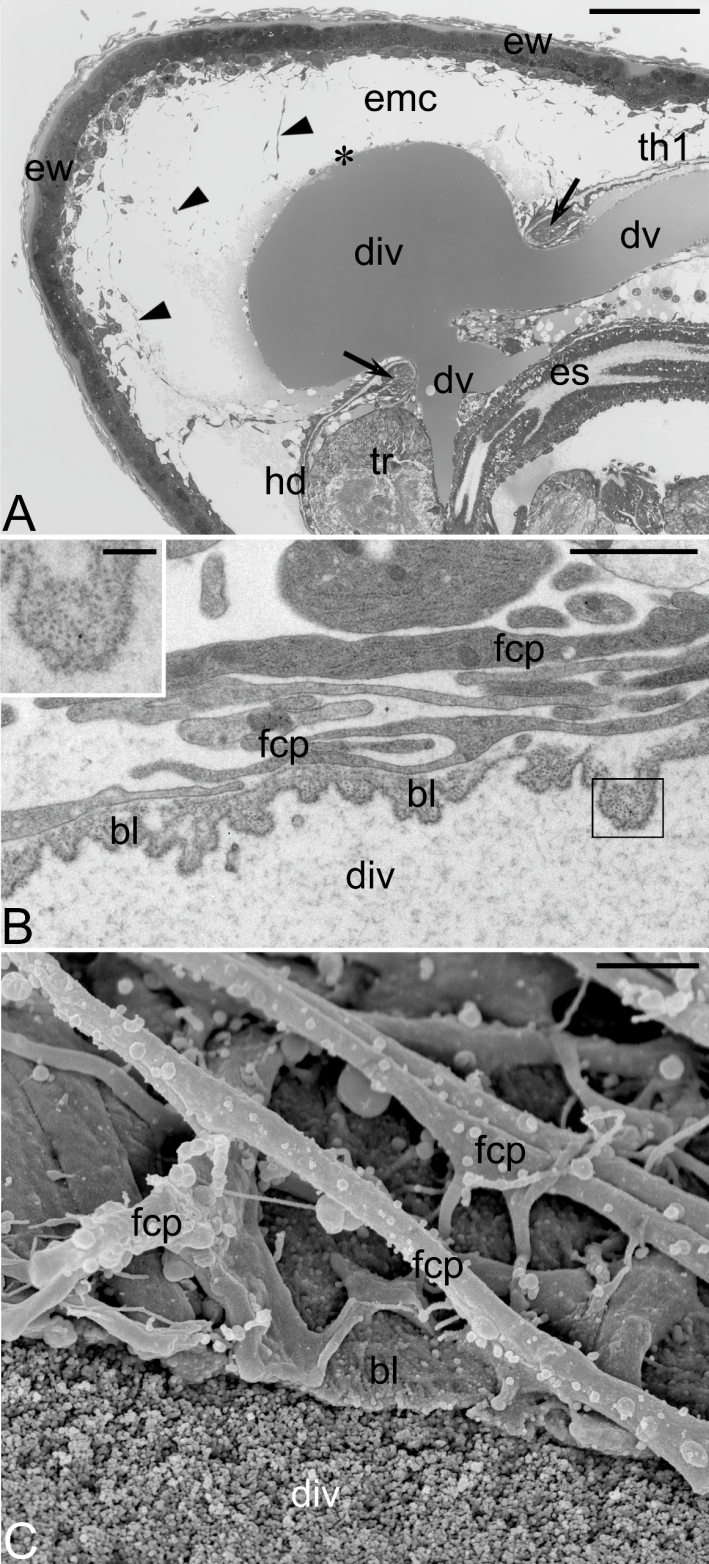
Cephalic vesicle. (A) Longitudinal section through the anterior region of the embryonic follicle containing a late embryo; note that the cephalic vesicle (asterisk) comprises a diverticulum (div) of the dorsal vessel (dv); the wall of the embryonic follicle (ew), invaginated body wall surrounding the cephalic vesicle (arrows); embryonic cavity (ec), follicular cell network present in the embryonic cavity (arrowheads), esophagus (es), head (hd), first thoracic segment (th1), tritocerebrum (tr); semithin section stained with methylene blue. Scale bar: 100 μm. (B) Transverse section through the wall of the cephalic vesicle; note basement lamina (bl) of the dorsal vessel and highly extended projections of the follicular cells (fcp) covering the lamina; magnified area (boxed in B) showing basement lamina filaments (inset). Scale bars: 2 μm, inset: 400 nm. (C) SEM micrograph of a fractured cephalic vesicle; basement lamina (bl), projections of the follicular cells (fcp), lumen of the dorsal vessel diverticulum (div) filled with haemolymph macromolecules. Scale bar: 5 μm.

During the late phase of the development, the embryo is characteristically ventrally bent: the head is oriented almost perpendicularly to the thoracic segments, whereas the tip of the abdomen and cerci are flexed anteriorly (Fig 3A and 3B). Our analyses have shown that the cephalic vesicle does not develop “at the occipital region of the head” as suggested by Heymons [9] but forms between the head and thorax (Fig 3B and 3C). In this region, the body wall is relatively thin and not completely sealed, leaving a single circular opening, through which a diverticulum (evagination) of the embryonic dorsal vessel extends outside the embryo (Fig 4A). The opening is encircled by a characteristic collar formed by an invaginated body wall (Fig 4A). Below the diverticulum (or anteriorly in the relation to the long embryo axis), the dorsal vessel divides into two vessels: one leads to the brain, the other opens directly to the haemocoel (Fig 4A). Concurrently to the formation of the cephalic vesicle, the follicular cells surrounding the anterior part of the embryonic cavity reorganize and move apart forming increasingly longer projections. As a result, a loose network of distant somatic (follicular) cells arises in contact with the head of the developing embryo. Significantly, some cells of this network associate with the dorsal vessel diverticulum, forming a layer on its surface (Fig 4B and 4C).

Our TEM analyses have shown that in contrast to the main part of the dorsal vessel, which is covered with muscle fibers, the wall of the vessel diverticulum comprises a basement lamina only (Fig 4B). This in turn indicates that only this constituent of the dorsal vessel wall bulges out of the embryo. The basement lamina of the diverticulum is built of parallel loosely arranged filaments (20–25 nm in diameter) (Fig 4B, inset), whereas its external surface is associated with the follicular cells and their elongated projections (Fig 4B and 4C). The latter form a lattice that firmly surrounds the vessel diverticulum (Fig 4C). It should be stressed that both elements constituting the wall of the cephalic vesicle are not tightly matted and presumably permeable which most probably enables free passage of molecules from the embryonic cavity to the haemocoel of the embryo. Above results indicate that the cephalic vesicle of *Hemimerus* consists of both ovarian (mother) and embryonic parts and, therefore, may be interpreted as a peculiar intraovarian analogue of placenta. We believe that this intriguing cephalic organ is involved in the transfer of low molecular weight compounds produced by metabolically active follicular cells and/or present in the embryonic cavity, directly into the circulatory system of the embryo. Here, the contractions of muscle cells encompassing the dorsal embryonic vessel may transport these compounds towards the brain and haemocoel. To our knowledge, similar structurally complex intraovarian organ integrating maternal (ovarian) and embryonic tissues, involved in embryo nourishment has never been reported.

In conclusion, our histological and TEM analyses have revealed unusual processes supporting nutrition during embryonic development of viviparous earwig, *Hemimerus talpoides*:

- Transfer of inactive (para-crystalline) ER stacks from the oocyte to the embryo and activation of these organelles as embryogenesis progresses.
- Plausible transfer of nutrients from the embryonic cavity directly to the circulatory system of the embryo, via the cephalic vesicle.

Both processes are apparently involved in the nourishment of the developing embryo and should be regarded as advanced traits related to the highly specialized reproductive strategy characteristic for *Hemimerus*. It is worthy to note finally that viviparous development of a closely related earwig, *Arixenia esau* is supported by entirely different morphological modifications and/or processes [14]. This, in turn, suggests that viviparity in hemimerids and arixeniids had evolved independently and that different organs/tissues had been employed in embryo nourishment during the evolvement of the matrotrophy in these dermapteran subgroups.
